# From Greenhouse Pest to Open-Field Invader: Widespread Distribution and Phenological Expansion of *Tuta absoluta* (Meyrick, 1917) in Temperate Hungary

**DOI:** 10.3390/insects17080850

**Published:** 2026-08-15

**Authors:** Klaudia Kőszegi, Attila Sándor Takács, Ágnes Balogh, Gergő Kovács, Viktória Szecskó, János Kutas, Annamária Tüh, Boldizsár Vörös, Ilona Tunyoginé Búzás, Miklós Bíró, Gábor Farkas, István Farkas, Kitti Marsai-Mikolics, Miklós Bozsó, Balázs Tóth, Antal Nagy

**Affiliations:** 1 Plant Protection and Soil Conservation Department, Major Department of Agriculture, Government Office of Fejér County, Ország Street 23, H-2481 Velence, Hungary; molyasz@mailbox.unideb.hu; 2 Institute of Plant Protection, Faculty of Agricultural and Food Sciences and Environmental Management, University of Debrecen, Böszörményi Street 138, H-4002 Debrecen, Hungary; 3 Department of Evolutionary Zoology and Human Biology, University of Debrecen, Egyetem Square 1, H-4032 Debrecen, Hungary; 4 Plant Protection and Soil Conservation Department, Major Department of Agriculture, Government Office of Pest County, Lehel Street 43-47, H-1135 Budapest, Hungary; 5 Department of Plant Protection Product and Fertilizer Assessment, Directorate of Plant Protection, National Food Chain Safety Office, Dayka Gábor Street 3, H-1118 Budapest, Hungary; 6 Plant Protection and Soil Conservation Department, Major Department of Agriculture, Government Office of Heves County, Kossuth Lajos Street 9, H-3300 Eger, Hungary; 7 Plant Protection and Soil Conservation Department, Major Department of Agriculture, Government Office of Zala County, Kinizsi Pál Street 81, H-8900 Zalaegerszeg, Hungary; 8 Plant Protection and Soil Conservation Department, Major Department of Agriculture, Government Office of Bács-Kiskun County, Deák Ferenc Square 3, H-6000 Kecskemét, Hungary; 9 Plant Protection and Soil Conservation Department, Major Department of Agriculture, Government Office of Csongrád-Csanád County, Rárósi Street 110, H-6800 Hódmezővásárhely, Hungary; 10 Plant Protection and Soil Conservation Department, Major Department of Agriculture, Government Office of Békés County, Szabolcs Street 34, H-5600 Békéscsaba, Hungary; 11 Plant Protection and Soil Conservation Department, Major Department of Agriculture, Government Office of Vas County, Sugár Street 9, H-9700 Szombathely, Hungary; 12 Plant Protection and Soil Conservation Department, Major Department of Agriculture, Government Office of Tolna County, Tormay Béla Street 18, H-7100 Szekszárd, Hungary; 13 Plant Health National Reference Laboratory, Directorate of Food Chain Safety Laboratories, National Food Chain Safety Office, Budaörsi Street 141-145, H-1118 Budapest, Hungary; 14 Lepidoptera Collection, Department of Zoology, Hungarian National Museum Public Collection, Hungarian National Museum, Baross Street 13, H-1088 Budapest, Hungary

**Keywords:** invasive pest, sex pheromone traps, tomato leaf miner, tomato, Gelechiidae, Solanaceae, open field

## Abstract

Invasive species pose increasing threats, particularly in agriculture. Here we assess the open-field distribution, population density, and phenology of *Tuta absoluta* (Lepidoptera: Gelechiidae), an invasive pest rapidly expanding its range into open-field habitats in Hungary. Countrywide monitoring using sex pheromone traps was conducted at 23 sites in 2023, complemented with detailed seasonal monitoring at two locations. The results reveal a wide open-field distribution of the pest in Hungary, occurring at 21 of the 23 sampled sites. Population densities were significantly higher in highly urbanised areas, suggesting that warmer climatic conditions enhance colonisation and population growth. Continuous adult flight activity was recorded from May to December, indicating prolonged reproductive activity and overlapping generations. Based on the degree-day requirements (approx. 460 °C days per generation), five generations per year were expected and detected at two intensively sampled sites. These findings demonstrate a marked shift from the “greenhouse pest” status toward successful colonisation of open fields, supported by increased overwintering potential. The observed expansion and phenological changes indicate that the climatic conditions of temperate Hungary are increasingly suitable for the persistence of *T. absoluta* populations outdoors. This study underscores the growing agricultural risk posed by *T. absoluta* in temperate regions and highlights the importance of early detection and integrated pest management strategies for mitigating its impact.

## Simple Summary

The South American tomato moth (*Phthorimaea absoluta* Meyrick, syn. *Tuta absoluta*) is a highly destructive pest of tomato crops and is increasingly spreading from greenhouse production into open-field environments. This study aimed to discover where this pest currently lives outdoors in Hungary, determine the size of its populations, and understand how it survives the changing seasons. Sex pheromone traps were used to detect the presence of the species in field tomato crops at 23 locations in Hungary, which was complemented with detailed seasonal monitoring at two specific locations. The results showed that the insect is present in almost all tested areas, with the highest numbers found in warmer cities. Furthermore, the moths actively fly and reproduce from spring to winter, producing approximately five generations in a single year. In conclusion, this insect has shifted from being a problem isolated to warm indoor greenhouses to a pest that successfully overwinters in open fields. This study provides vital value to society by warning farmers and home gardeners about the expanded outdoor threat. By understanding the insect’s new outdoor survival habits, agricultural workers can detect the pest earlier and develop better methods to protect their tomato plants, thereby securing local food production against a growing risk.

## 1. Introduction

Invasive species have multifaceted negative effects on native biodiversity, causing problems for nature conservation, agriculture, and forestry at the same time. Additionally, they can be harmful especially in urbanised areas, causing health risks and several types of nuisances for the human population [1]. The economic loss caused by alien species is significant worldwide and has continuously increased for the last few decades [2,3]. In the European Union, the costs of protection against them add up to more than 100 million Euros per year [4].

Protection against invasive species is more effective in the early phase of invasion rather than after their full colonisation [5]; thus the early detection of their emergence has a key role in their management. Investigations of their biology; ecology, including plant–insect, prey–predator and host–parasite and multilateral interactions; chemical ecology; host plant and habitat preferences; etc., help us to understand the mechanisms of invasion, colonisation and damage and develop new innovative methods that act against them [6].

*T. absoluta* is a Gelechiidae pest that originated from South America and is native to Argentina, Bolivia, Brazil, Chile, Colombia, Ecuador, the Galapagos Islands, Paraguay, Peru, Uruguay and Venezuela [7,8]. It has been seen as a pest species since 2006 when it was introduced into Europe [9,10]. Its occurrence has been proven in more than 35 European countries, spanning a wide geographical range from the Mediterranean Basin to Northern Europe (e.g., Norway, Sweden, Great Britain) and Eastern Europe (e.g., Russia, Ukraine, Belarus) [11,12,13,14,15]. The expansion of the pest was extremely rapid in the Mediterranean region due to the optimal environmental conditions, but it also spread toward temperate zones using artificial habitats as greenhouses and foils where they can overwinter even under extreme climatic conditions [16,17,18]. The development, survival, reproduction, and longevity of *T. absoluta* are strictly temperature-dependent [19]. In its native range and in invaded tropical or subtropical regions, the pest can produce up to 10–12 generations per year. Under greenhouse conditions, optimal temperatures allow for continuous reproduction, covering the whole phenology of the host plant and increasing yield loss [20]. Degree-day models indicate that the species requires approximately 460 °C days to complete a single generation, which limits its outdoor proliferation in colder climates but allows temporary local populations to establish during the summer [21]. Data on the distribution in Hungary were reported by Fazekas and Szeőke (2011) [22] and Ágoston and Fazekas (2014) [23].

The main cultivated host plant of the pest is tomato (*Lycopersicon esculentum* (L. H. Karst.)), but it can also feed on other cultivated and wild hosts such as green pepper (*Capsicum annuum* L.), potato (*Solanum tuberosum* L.), sweet cucumber (*Solanum muricatum* Ait.), black nightshade (*Solanum nigrum* L.), wild tobacco (*Nicotiana rustica* L.), tobacco (*Nicotiana tabacum* L.), Jimsonweed (*Datura stramonium* L.), Chilean boxthorn (*Lycium chilense* Bertero), and Chinese wolfberry (*Lycium barbarum* L.) [24].

Although tomato has a diverse species-rich pest assemblage, especially in greenhouses, including harmful pests such as *Meloidogyne* spp., *Frankliniella occidentalis*, *Trialeurodes vaporariorum*, *Bemisia tabaci*, *Liriomyza* spp., *Aculops lycopersici*, and *Tetranychus urticae*, the appearance of *T. absoluta* could further aggravate the situation [25,26].

The economic impact of *Tuta absoluta* is exceptionally severe. Without adequate control measures, the pest can damage leaves, stems, and fruits, leading to 80–100% yield loss in both greenhouses and open-field tomato crops [16,17,27]. Its rapid spread across Europe has drastically increased plant protection costs, forcing farmers to increase insecticide use alongside other IPM tools, such as mass trapping, mating disruption with sex pheromones, and biological control agents like the predatory bug *Macrolophus pygmaeus*. Furthermore, managing *T. absoluta* is becoming increasingly difficult as the species rapidly develops resistance to frequently used insecticides, such as pyrethroids and spinosad. This quick resistance makes pest control more expensive and creates trade risks, since farmers must stay within strict European pesticide limits (MRLs) [28,29,30,31,32,33].

Effective IPM technologies against tomato pests including *T. absoluta* require actual data on the appearance, swarming and even biology of the pest, changing during the adaptation of the local population, derived by climatic changes and the effects of treatments.

In the temperate zone, the range of suitable habitats and parallelly the spatial distribution of the species are limited. According to our knowledge so far, *T. absoluta* can overwinter mainly in artificial sites and has only temporary local populations in open fields, where the species must continuously recolonise after frost periods [21]. However their early detection in February and March in open fields suggests their potential for overwintering in wild habitats as well [23].

In line with climate change, the question arises of whether the open-field distribution of the species and its phenology and overwintering ability will change in temperate regions. Therefore, the primary goal of this study was to assess the open-field distribution and population dynamics of *T. absoluta* across Hungary using sex pheromone traps in 2023. We tested the following hypotheses: (1) local population densities are positively correlated with the level of urbanisation due to the urban heat island effect, proving the existence of warmer microclimates, and (2) current weather conditions in Hungary provide sufficient accumulated degree-days for the pest to complete multiple overlapping generations and potentially overwinter in open fields.

## 2. Materials and Methods

In 2022, preliminary studies were conducted in urbanised and suburban (with extensive horticultural and agricultural sites) areas in Gyula and Békéscsaba-Gerla (Békés County). The sampling sites were small extensively managed tomato fields (at least 50 m^2^) with minimal use of synthetic pesticides in house gardens.

**Figure 1:**
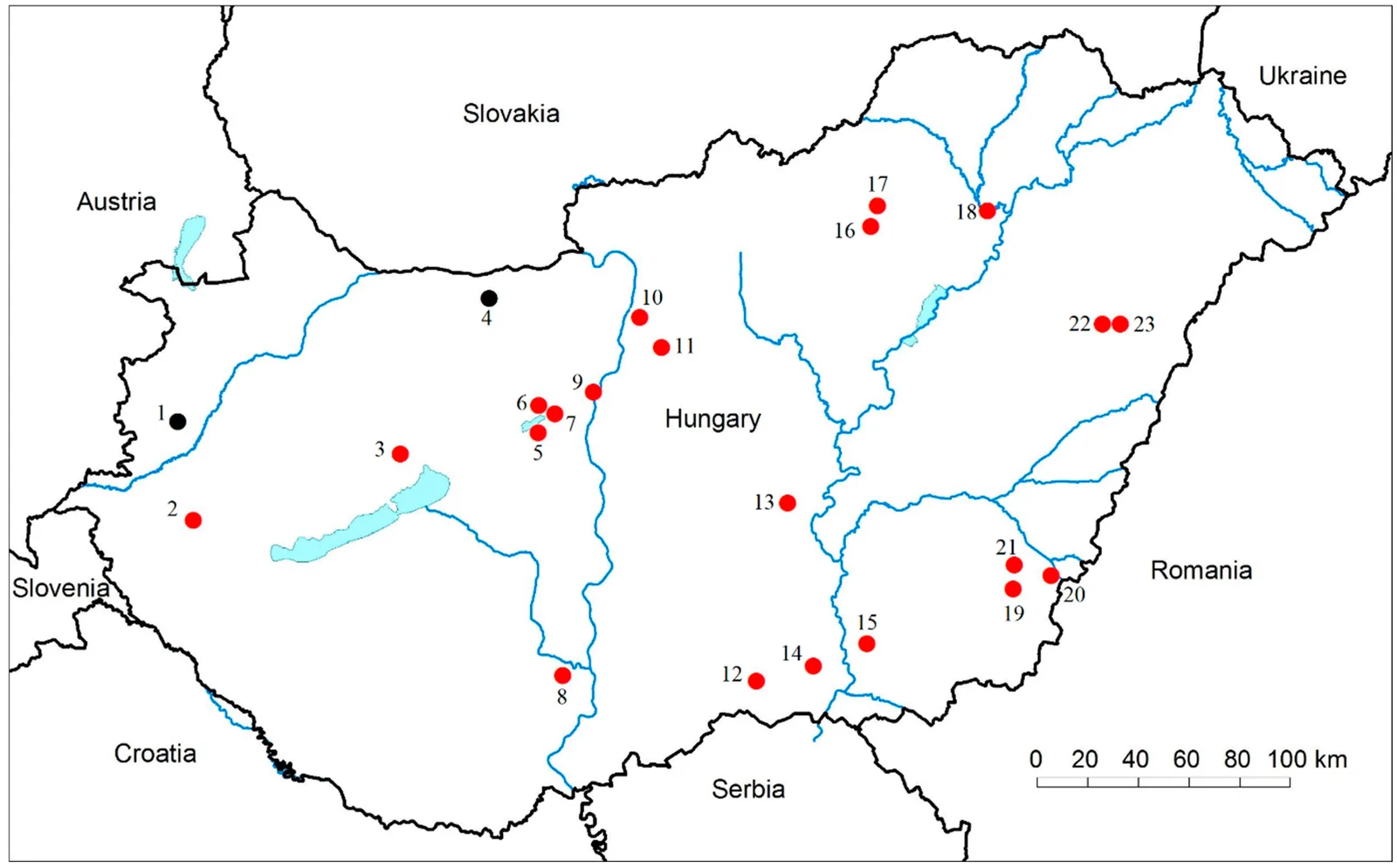
The sampling sites studied during the countrywide monitoring of *Tuta absoluta* in 2022 and 2023. Red dot: Site where traps caught the target species. Black dot: Sites without catches.

Countrywide samplings were carried out in 2023 at 23 sampling sites in total (Table 1, Figure 1). The sampling sites were in similar habitats to those sampled in the preliminary study. At two sites, Budapest and Gerla, the samplings were conducted from 1 May to 30 November 2023, with a daily check performed on the traps. At other sites (*n* = 21), samplings were started in September, and the traps were employed for a month until the baits were exhausted, with the exception of Százhalombatta, where the trap worked from 1 July to 31 August.

Trappings were carried out with delta-type sticky traps baited with the sex pheromone of the targeted species (major component: (3E,8Z,11Z)-3,8,11-tetradecatrienyl acetate [34]) produced by Csalomon^®^, Budapest, Hungary. This method was chosen due to its high capture capacity and low field maintenance requirements, which make it reliable for large-scale, extensive, multisite outdoor experiments [35,36].

One trap was hung on a stable place (on shoots of the plants or stakes) at a height of 1.5 m at each sampling site among tomato plants. In Békéscsaba-Gerla and Budapest, where population dynamics were monitored, the baits were replaced after four-week periods. Sticky sheets were replaced depending on the number of moths caught, before the covering by captured specimens decreased the efficiency of the trap.

In the case of Békéscsaba-Gerla and Budapest, the daily number of individuals caught was recorded. The daily minimum and maximum temperatures were obtained from the closest validated meteorological stations of the MetNet database to calculate the mean daily temperatures accurately. In other cases, the density of the local moth population was categorised as follows: 0= no catches; 1 = low density (<50 moths in the trap); 2 = medium density (51–100 moths in the trap); 3 = high density (>100 moths in the trap). Sites were characterised by habitat type and the level of urbanisation (as small, medium or high) (Table 1). Urbanisation levels were classified based on local population size and density: small (villages and small towns, <10,000 inhabitants), medium (medium-sized towns, 10,000–100,000 inhabitants), and high (large cities, >100,000 inhabitants).

The effect of urbanisation on pest density was analysed using a comparison of the mean density values of the differently urbanised sites with a Mann–Whitney U-test. For the analysis, the Statsoft Statistica 7 program package was used.

Phenology and development were evaluated using temperature threshold (8 °C) and degree-day per generation (460 °C days) data published and used by Desneux et al. (2010) [16] and Tonnang et al. (2015) [37]. Daily mean temperatures were calculated using the daily minimum and maximum temperatures measured by MetNet.

## 3. Results

### 3.1. Distribution and Densities of Pest

**Figure 2:**
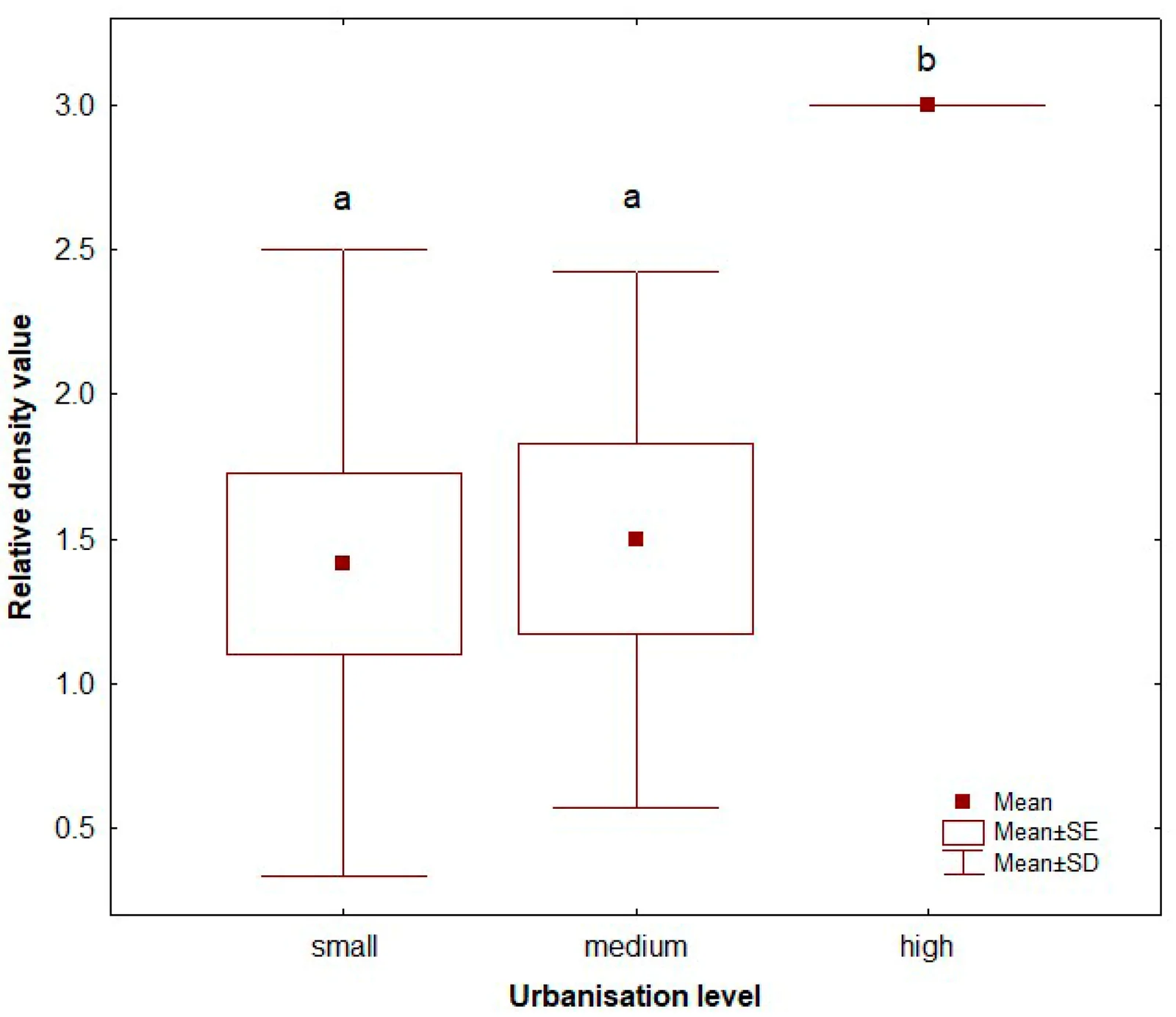
Mean relative frequencies of *Tuta absoluta* populations at sites with different levels of urbanisation. Small letters show significant differences based on Mann–Whitney U-test (small–medium: *p* = 0.792; small–high: *p* = 0.037; medium–high: *p* = 0.034).

The pest was recorded at 21 of the 23 sampled sites, proving the wide distribution of the species in open fields across Hungary (Table 1, Figure 1). The pest occurred in nearly all regions except northwestern ones, but increasing the sampling effort may provide new distribution data even in this region. Additionally, data on the southwestern (Transdanubian hilly region) and northeastern regions (Bereg and Szatmár regions) remain mainly unknown, and these areas are designated the priority of further investigations (Figure 1 and Table 1). In large cities (Budapest and Debrecen), the abundance of pest populations was generally higher than in small- and medium-sized towns and villages. The difference between the highly urbanised sites and less urbanised ones was statistically significant (Figure 2).

### 3.2. Phenology of Pest in Open Field

**Figure 3:**
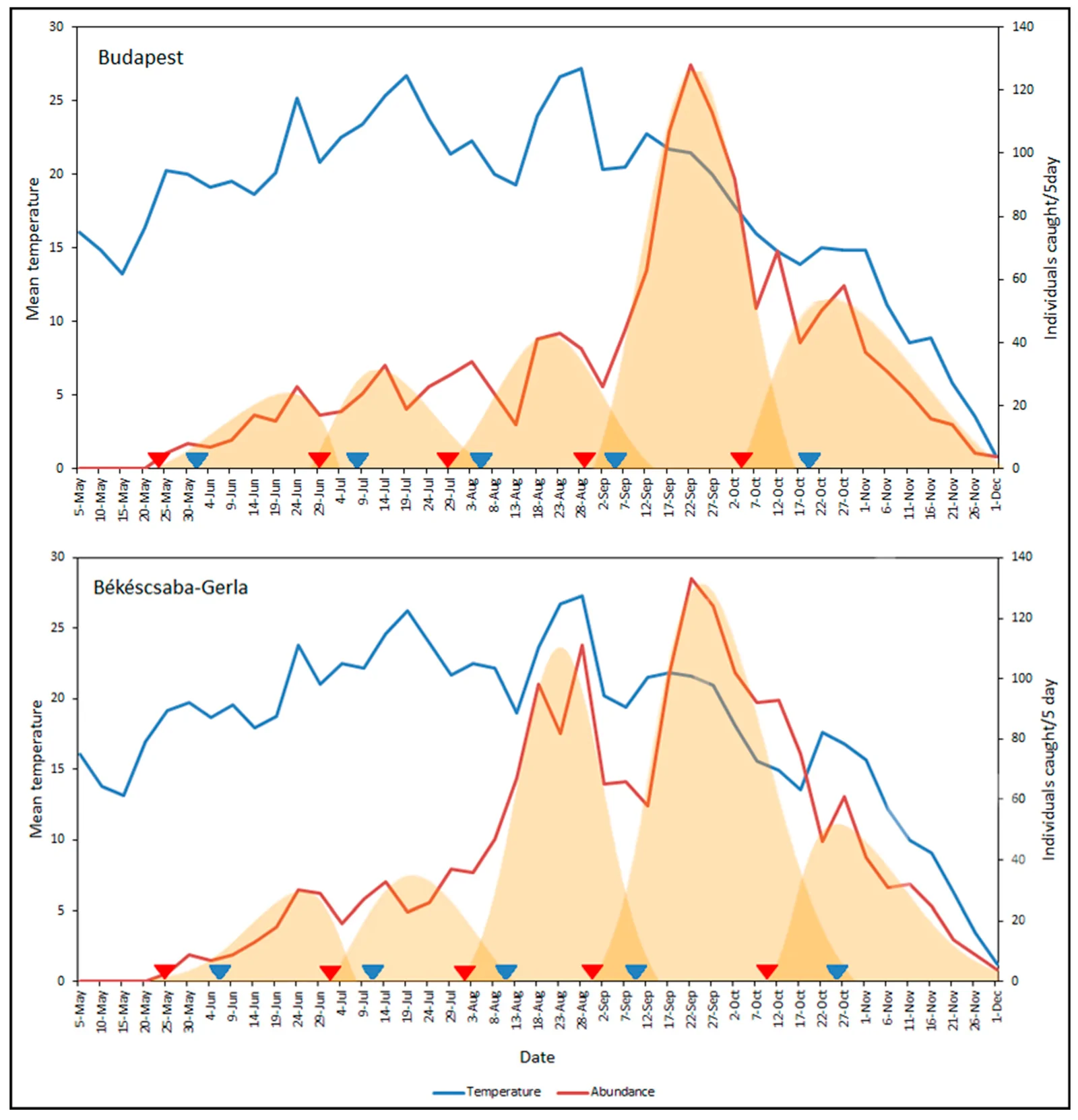
Temporal changes in five-day catches of *Tuta absoluta* and five-day mean air temperature at Budapest and Békéscsaba-Gerla sampling sites in 2023. Red signs: Recorded start of flight of 1st gen. and calculated dates of further gens. Blue signs: Calculated start of gens. Yellow shading indicates potential span of consecutive overlapping generations based on degree-day values and sampled swarming dynamic.

*Tuta absoluta* imagoes were on the wing throughout the entire sampling period. Based on the temperature threshold and degree-day requirements per generation, the calculated starting dates of the first-generation swarm should have been the 1 and 6 June 2023 in Budapest and Békéscsaba-Gerla. However, the swarming started one or two weeks earlier, on the 24 and 25 May 2023 at the two sites, and consecutive generations flew until early December (Figure 3). The calculated numbers of generations were 5.3 and 5.2 in Budapest and Békéscsaba-Gerla respectively. In Békéscsaba-Gerla, located south of Budapest in southeast Hungary, the total degree-day values in the whole year were slightly lower (2392.6 °C days) than in Budapest (2426.7 °C days), but during the flight period of the pest, they was nearly the same at the two sites (2061.4 and 2060.2 °C days). The pest grew by five generations at both sites. Between the recorded and calculated dates when the consecutive generations started flight, there was a week and even two weeks that elapsed in the case of the fifth generation (Figure 3).

In the urbanised habitats studied in Budapest, the number of captured individuals continuously increased during the flight of the first three overlapping generations. Subsequently, in early September, the fourth generation showed a substantial outbreak with a peak on the 22 of September, when the 5-day catches reached 120 individuals (Figure 3).

In Békéscsaba-Gerla, the number of individuals caught increased throughout the flight of the first two generations, and the next two ones showed similar outbreaks with 5-day catches higher than 100 and 120 individuals. The decrease in the catches was similar to that in Budapest from early October to December (Figure 3).

## 4. Discussion

During sampling, sex pheromone traps proved their efficacy [35,38,39]. Since the last assessment of the *Tuta absoluta* populations in Hungary [23], their distribution has radically changed. The former greenhouse pest now shows wide distribution in open fields and causes significant damages in both horticultural lands and house gardens, where its main host, tomato, is one of the most popular cultivated plants. The pest was absent from sampling sites in northwest Hungary, but further sampling might reveal its presence. Future monitoring should also focus on the uninvestigated southwestern and northeastern regions.

The density of local populations highly depended on the habitat. Highly urbanised sites in large cities showed higher abundances than less urbanised ones. This is likely driven by the urban heat island (UHI) effect. The thermophilic character of the moth formerly limited its open-field distribution and overwintering success [21,40,41,42]. However, higher temperatures in urbanised areas allow for faster development, increased reproductive rates, a higher number of overlapping generations per year, and higher overwintering success. Similar expansion and overwintering success driven by the UHI effect have been already observed in other invasive pests in Europe, such as *Halyomorpha halys* and *Nezara viridula* [43,44]. Our findings suggest a distinct change over the last decade: the overwintering success of *T. absloluta* under open-field conditions, particularly in urban microclimates, has increased. However, to prove this assumption, further targeted tests are needed.

The swarming dynamics of the two regularly sampled populations were similar to those observed in Bulgaria, where the potential number of generations per year varied between 3.9 and 5.1 [37,45]. Based on our local temperature data, the potential number of generations was 5.1 and 5.2. Catches showed five overlapping generations at both sites. Moths started their flight earlier than predicted by the temperature data.

Our results suggest that the habitat use, distribution, number of generations per year and even overwintering status of *Tuta absoluta* have gone through dramatic changes since its introduction into the Carpathian Basin in 2010. The pest has become widely distributed in open fields and grows by 4–5 generations per year. Its early swarming in May suggests its successful overwintering in open fields. These changes may be explained partly by the effect of climate change and the adaptation of local populations. The warmer microclimates of habitats, e.g., in urbanised sites, support its population growth and help its spread and adaptation in the temperate zone. Our findings highlight the increasing damage risk and economic importance of the species, proving the need for further studies and the careful management of affected cultures.

## 5. Conclusions

This study highlights that the distribution and phenology of *Tuta absoluta* have changed since its introduction into the Carpathian Basin. The pest shifted from being primarily a greenhouse pest to a widespread open-field invader, successfully completing up to five generations a year in Hungary.

Our findings suggest that urbanisation through the UHI effect plays a key role in helping the pest colonise and increase its population outdoors. Additionally, the early flight of adults in May strongly indicates that the species may successfully overwinter under the open-field conditions of the temperate zone.

## Figures and Tables

**Table 1:** The sampling sites studied in the countrywide monitoring of *Tuta absoluta* in 2022 and 2023 with the coordinates, pest density (0 = no catches; 1 = low density (<50 moths in the trap); 2 = medium density (51–100 moths in the trap); 3 = high density (>100 moths in the trap)), level of urbanisation (small, medium, large) and relative density value of the pest (0 = no catches; 1 = low; 2 = medium; 3 = high).

No.	Site	GPS (N/E)	Pest Density	Level of Urbanisation
1	Tanakajd	47°11′15.8′/16°44′20.3″	0	small
2	Zalaegerszeg	46°50′02.4″/16°51′29.3″	1	medium
3	Veszprém-Gyulafirátót	47°08′36.2″/17°56′56.9″	1	medium
4	Baj	47°39′05.4″/18°21′31.0″	0	small
5	Gárdony	47°11′42.6″/18°37′31.2″	1	medium
6	Velence	47°14′37.0″/18°38′37.5″	1	small
7	Kápolnásnyék	47°14′43.0″/18°40′55.3″	1	small
8	Őcsény	46°18′50.7″/18°45′08.6″	1	small
9	Százhalombatta	47°18′52.9″/18°55′26.5″	1	medium
10	Dunakeszi	47°37′37.2″/19°08′31.6″	1	medium
11	Budapest	47°30′02.3″/19°17′04.8″	3	high
12	Ruzsa	46°17′10.1″/19°45′20.4″	3	small
13	Szentkirály	46°55′04.8″/19°55′42.6″	1	small
14	Szatymaz	46°20′28.9″/20°02′41.2″	3	small
15	Hódmezővásárhely	46°25′20.4″/20°16′31.9″	3	small
16	Eger	47°53′52.0″/20°22′51.0″	1	medium
17	Felsőtárkány	47°57′57.9″/20°24′33.3″	1	small
18	Sajószöged	47°56′57.0″/20°59′30.6″	1	small
19	Szabadkígyós	46°35′59.5″/21°05′04.8″	2	small
20	Gyula	46°38′28.6″/21°16′08.1″	3	medium
21	Békéscsaba-Gerla	46°42′18.3″/21°10′56.6″	3	medium
22	Debrecen	47°33′09.0″/21°36′06.6″	3	high
23	Debrecen	47°31′35.4″/21°40′56.4″	3	high

## Data Availability

The original contributions presented in this study are included in the article. Further inquiries can be directed to the corresponding authors.

## Abbreviations

The following abbreviations are used in this manuscript:

KK	Klaudia Kőszegi
AST	Attila Sándor Takács
AN	Antal Nagy
ÁB	Ágnes Balogh
GK	Gergő Kovács
VS	Viktória Szecskó
JK	János Kutas
AT	Annamária Tüh
BV	Boldizsár Vörös
ITB	Ilona Tunyoginé Búzás
MB1	Miklós Bíró
GF	Gábor Farkas
IF	István Farkas
KMM	Kitti Marsai-Mikolics
MB2	Miklós Bozsó
BT	Balázs Tóth
